# Reemerging of Encephalomyocarditis Virus in Pigs in Brazil: Pathological and Viral Characterization

**DOI:** 10.1155/2023/6582778

**Published:** 2023-12-07

**Authors:** Anderson H. Gris, Raquel S. Alves, Laura J. Camargo, Letícia F. Baumbach, Jean C. O. Menegatt, Emanoelly M. S. Silva, Fernanda F. Perosa, Rafael P. Lima, Marianna Bertolini, Aparecida T. L. Fiúza, Tatiane T. N. Watanabe, Cláudio W. Canal, David Driemeier

**Affiliations:** ^1^Faculdade de Veterinária, Setor de Patologia Veterinária, Universidade Federal do Rio Grande do Sul, Porto Alegre, Brazil; ^2^Faculdade de Veterinária, Laboratório de Virologia, Universidade Federal do Rio Grande do Sul, Porto Alegre, Brazil; ^3^Boehringer Ingelheim Animal Health Brazil, São Paulo, Brazil; ^4^Department of Population Health and Pathobiology, College of Veterinary Medicine, North Carolina State University, Raleigh, NC, USA; ^5^Antech Diagnostics, West Olympic Boulevard, Los Angeles, CA, USA

## Abstract

Encephalomyocarditis virus (EMCV) is a zoonotic disease caused by a highly pathogenic virus that affects wild and domestic animal species, with rodents as its reservoir. Most recently, in South America, this virus was detected in an outbreak affecting humans in Peru. In Brazil, EMCV infection was described in some wild species, in horses, and once in farrowing pigs. The aim of this study is to report the reemergence of EMCV in commercial growing–finishing pigs from two different farms that experienced sudden pig death in midwest Brazil. This aim was achieved through gross pathology, histologic examination, RT‒PCR analysis, and genetic characterization of the virus. Clinical signs, such as trembling, dyspnea, and squealing sounds shortly before death, were only occasionally observed and were nonspecific. On gross examination, cardiomegaly was observed, along with multifocal pale tan foci in the epicardium extending to the myocardium on the cut surface. Microscopically, there was severe myocardial necrosis, dystrophic mineralization, fibrosis, and lymphoplasmacytic and histiocytic myocarditis. Gross and microscopic examinations of the rats were unremarkable. The RT‒PCR analyses of the pig and rat organs were positive for EMCV, and the phylogenetic analysis of the VP1 gene showed that the samples from pigs and rats contained similar strains that had their closest relatives identified in humans in Peru. This is the first genetic characterization of EMCV in Brazil, and the other findings confirm the reemergence of the disease that was transmitted from rats to pigs.

## 1. Introduction

The *Picornaviridae* family includes the genus *Cardiovirus*, which comprises six species labeled from A to F. Specifically, *Cardiovirus* A includes the encephalomyocarditis virus (EMCV), which is further divided into two serotypes: EMCV 1, EMCV 2, and a putative third strain (EMCV 3) [1, 2]. EMCV has a worldwide distribution, with rats and mice serving as the reservoir species responsible for the spreading of the virus and causing outbreaks in susceptible animals. The susceptible animals predominantly comprise wild or domestic mammals, with livestock animals, particularly swine, being of interest [3].

The first documented outbreak of EMCV in swine occurred in Panama in 1958, followed by occurrences in multiple countries across North America, Europe, Asia, and Oceania [4, 5]. In South America, there have been reported cases involving humans in Peru, with viral isolation and sequencing, as well as detection of antibodies against EMCV [6]. Regarding animals in Peru, there are only descriptions of serological evidence indicating the presence of the virus in rodents [7].

In Brazil, EMCV infections have been previously reported in mice, wild animals (birds, *Proechimys guyannensis*, and *Marmosa* sp.), horses, and some mosquito species [8]. However, concerning pigs, there is only one documented case in pigs in 1985, where 9 out of 10 piglets from a single litter died in southern Brazil [9]. It is worth noting that these studies from the 1960s to 1980s did not involve molecular techniques, so there was no information available regarding the genetic characteristics of EMCV in Brazil. Furthermore, no additional outbreaks have been documented in the country since then. The aim of this study is to report the reemergence of EMCV causing sudden death in growing–finishing pigs in Brazil.

## 2. Materials and Methods

Two growing–finishing farms located in the state of Mato Grosso in the Midwest region of Brazil, experienced outbreaks of sudden death in pigs between 120 and 130 days old. Clinical and epidemiological data were collected during farm visits in April 2023. Postmortem examinations were conducted on 7 dead pigs (*Sus scrofa domesticus*) and 10 rats (*Rattus rattus*). Gross lesions were documented, and samples were collected from major organs (brain, heart, lungs, liver, kidneys, spleen, large intestine, small intestine, lymph nodes, and stomach) and preserved in 10% neutral buffered formalin. Additionally, samples of lymph nodes, brain, heart, and liver from pigs, along with brain, spleen, heart, and rectal feces samples from necropsied rats, were frozen and stored at −80°C. The formalin-fixed samples were trimmed, routinely processed, embedded in paraffin wax, and sectioned into slices measuring 3–4 *µ*m in thickness. These slices were then mounted on glass microscope slides and routinely stained with hematoxylin and eosin (HE). Afterward, the slides underwent a routine histopathological examination to characterize the microscopic findings.

The frozen fragments from both the swine and rats were macerated using phosphate-buffered saline (PBS) (pH 7.2) and screened for the presence of EMCV using an RT‒PCR protocol [10]. Additionally, the feces from the rats were subjected to analysis. The rat samples were assessed in pools of samples, while the swine organs were screened individually, except for four samples of liver that were tested in pool. RNA isolation was performed using TRI Reagent® (Sigma‒Aldrich, St. Louis, MO, USA). Complementary DNA (cDNA) synthesis was performed using M-MLV Reverse Transcriptase (Invitrogen, Carlsbad, CA, USA) with random primers (Invitrogen, Carlsbad, CA, USA). RT‒PCR targeting a 286-bp fragment of RNA-dependent RNA polymerase (RdRp) using Invitrogen Taq platinum (Thermo Fisher, Carlsbad, CA, USA) was utilized to ascertain which organs contained *Cardiovirus A* in each species. The positive samples underwent a new RT‒PCR to amplify 500-bp of the viral protein 1 (VP1) gene of the virus [11] for phylogenetic analyses, following ICTV guidelines [12]. The resulting amplicons were purified using the PureLink™ Quick PCR Purification Kit (Invitrogen), followed by bidirectional DNA sequencing using an ABI PRISM 3100 Genetic Analyzer along with the 238 Big Dye Terminator v.3.1 cycle Sequencing Kit (Applied Biosystems, USA). Later, consensus sequences were assembled using Geneious software (version 2023.1.1).

To determine the closest related sequences to those obtained in this study, nucleotide sequences were compared against the GenBank database using the BLASTN and BLASTX programs, available at https://blast.ncbi.nlm.nih.gov/blast.cgi. Sequences with the best hits were obtained along with the reference sequences from species *A*, *B*, *C*, *D*, *E*, and *F* of the *Cardiovirus* genus and aligned using ClustalW with MEGA6 software [13]. Pairwise genetic distances were calculated by p-distance, and nucleotide phylogenetic trees were constructed using maximum likelihood (ML) inference with the HKY + G method (VP1) in 1,000 bootstrap replicates.

## 3. Results and Discussion

Two commercial farms (Farms A and B) that house 4,500 pigs each and that are operated by a swine company located in the midwestern state of Mato Grosso, Brazil, have faced sudden death outbreaks in growing–finishing pigs since October 2022. Upon clinical examination, some pigs presented occasional and nonspecific clinical signs characterized by trembling, dyspnea, and squealing sounds just before succumbing to death. The deaths occurred throughout the day, with a higher concentration observed during the later hours of the afternoon and at night. Pigs from multiple pens of both sexes, and mainly the largest animals in the batch, were affected. The mortality rate ranged between 9% and 10% in the affected batches, with peaks of 50 deaths per day. At the onset of the increase pig mortalities, field veterinarians implemented antipyretic therapy (dipyrone) and antibiotic treatments (florfenicol, trimethoprim sulfamethoxazole, tiamulin, and amoxicillin). Unfortunately, these interventions proved unsuccessful in reducing pig deaths. During farm visits and facility inspections, rats were seen inhabiting the facilities and vicinities. The rodent control measures implemented on the farm, including chemical control (Farms A and B) and traps (Farm A), had failed, which allowed the collection of 10 dead rats for necropsy.

**Figure 1:**
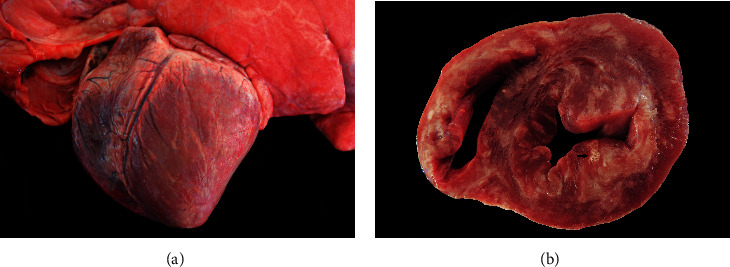
Gross lesions of pigs naturally infected by EMCV. (a) Heart: the surface of the epicardium and myocardium presents multifocal to coalescent areas of 0.5–1 cm in diameter or in a linear pattern with a pale tan discoloration on both sides of the ventricle. Pig 2, Farm B. (b) Heart cross-section: nearly 80% of the myocardium of both atria, ventricles, and interventricular septa have multifocal to coalescent pale tan discoloration foci measuring 0.3–1 cm in diameter with occasional opaque white areas (arrow) measuring up to 0.5 cm in diameter. Microscopically, the latter corresponds to dystrophic mineralization. Pig 3, Farm B.

Five necropsies were performed in pigs from Farm A and two from Farm B. All pigs exhibited good body condition scores and had died suddenly. The macroscopic examination of the pig hearts revealed varying degrees of enlargement, with transmural multifocal areas of pale tan discoloration ranging from 0.5 to 1 cm in diameter or occurring in a linear pattern and extending to the myocardium (Figures 1(a) and 1(b)). Mild hydropericardium (7/7) and mild pulmonary edema (4/7) were also observed. In one pig, as well as in all other organs examined in the 10 rats, no significant alterations were observed during the gross examination.

**Figure 2:**
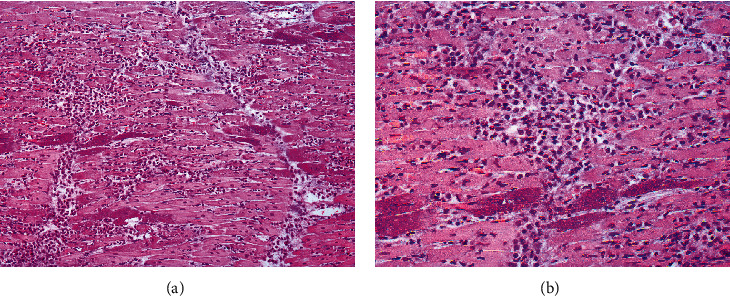
Microscopic findings of the myocardium of pigs naturally infected by EMCV. (a) Myocardium showing severe multifocal to coalescent inflammation associated with proliferation of fibrous connective tissue (fibrosis) and some areas of intense basophilic material (mineralization) are observed. Pig 2, Farm B, hematoxylin and eosin (HE), 200x. (b) Higher magnification of the myocardium demonstrating the inflammatory infiltrate of lymphocytes, plasma cells, and macrophages with fibrosis and mineralization. Pig 2, Farm B, HE, 400x.

The histopathological examination revealed myocardial lesions in all pigs, with these lesions being characterized by myocardial necrosis (4/7) associated with a mild to severe inflammatory infiltrate of lymphocytes, plasma cells, and macrophages surrounding necrotic myocardial fibers and blood vessel (7/7). In some areas, in addition to the inflammatory infiltrate, there was also mineral deposition (4/7) and mild proliferation of fibrous connective tissue (4/7) (Figures 2(a) and 2(b)). In the lungs, the alveoli were filled by peracute to acute edema (4/7). The liver had diffuse congestion (2/7). No significant microscopic changes were observed in the other evaluated organs of the pigs or in any examined organs of the rats.

A total of 17 samples were analyzed by RT‒PCR against EMCV, comprising 10 samples from rats and 7 from swine (Table S1). Of the two pools of samples obtained from the rats, one pool consisting of brain tissue and another with feces tested positive for the RT‒PCR protocol targeting the RdRp of EMCV. Regarding the swine samples, the liver (pool of four), lymph node (2/3), and heart (2/3) were also tested positive by RT‒PCR. Lymph node and heart from one swine and brain from another tested negative.

**Figure 3:**
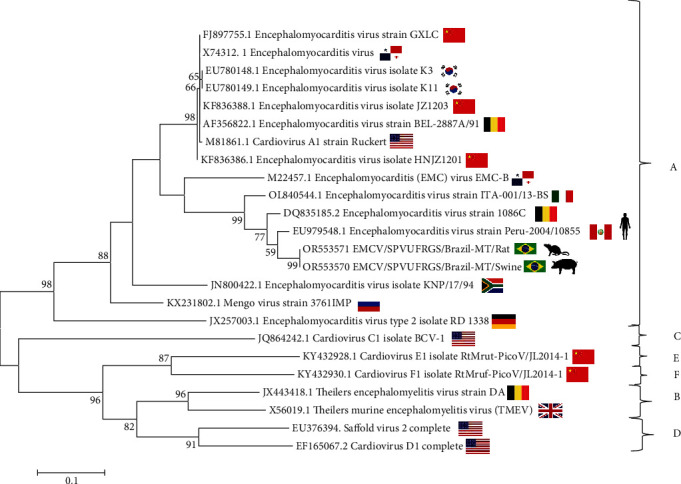
Nucleotide tree constructed using sequences of the VP1 gene. Phylogenetic reconstruction based on VP1 sequences of EMCV. The phylogenetic tree was constructed with the MEGA 6 software using the maximum likelihood algorithm method based on the HKY + G model in 1,000 replicates. The letters at the right extremity represent the species of the genus *Cardiovirus*. The sequences obtained in the present study aligned within the species *Cardiovirus A* and were deposited in the GenBank database under accession numbers OR553571 and OR553570.

Two samples were selected for phylogenetic analysis of the partial VP1 region, one from the pool of rat brain tissue and another from a swine heart. The sequences obtained from EMCV/SPVUFRGS/Brazil-MT/Rat (accession number OR553571) and EMCV/SPVUFRGS/Brazil-MT/Swine (accession number OR553570) were consistently grouped together with other members of the *Cardiovirus* lineage l A (Figure 3). This clustering was supported by a bootstrap value of 100. The rat and swine sequences clustered within a closer branch, sharing a terminal node with a strain previously identified in a human from Peru (accession number EU979548.1) [6], in a rat (accession number DQ835185.2) [14] and swine (accession number AJ617360.1) [15] from Germany, and with a swine strain from Italy (accession number OL840544.1) [11].

The diagnosis of EMCV infection was established through the association of clinical, epidemiological, macroscopic, microscopic, and molecular findings. In Brazil, there have been two previous occurrences of EMCV. The first occurred in 1962, affecting mice, wild animals (*Proechimys guyannensis* and *Marmosa* sp.), horses, birds, and some mosquitoes from the northern region of the country. The second occurred in 1985 and was characterized by an outbreak in a batch of farrowing pigs from the southern region of the country. However, after that occurrence, no further diagnoses of this disease were documented in the country [8, 9]. In Brazilian territory, there was a gap of 38 years between the last EMCV infection report and the present report, which occurred in the midwestern region of the country and was located far from the previous report. Additionally, the absence of the diagnosis of EMCV infection may be linked to the absence of the virus in the country or due to negligence in the diagnosis. However, to confirm any of these scenarios, further studies involving serological exams are needed.

More recently, in South America, more specifically in Peru, EMCV infections were reported in two febrile humans, along with serological detection in humans and rodents [6, 7]. Brazil shares a border of nearly 3,000 km with Peru, which may facilitate animal and road traffic that enhances the circulation of the virus between the two countries [16]. This is underscored by the similarity observed between our sequences and those reported in Peru, as illustrated in Figure 3 [6].

Furthermore, the human cases of EMCV infection reported in Peru highlight the importance of the virus as a zoonotic agent [6]. Given the similarity of the genetic sequences between the work in humans and the present report, it underscores the importance of the continuous surveillance in the diagnosis of animals to characterize the agents circulating in the country. These agents can potentially affect other species, including humans, as seen with EMCV. Therefore, through this surveillance, it becomes possible to detect emergence or reemergence of infectious diseases. These occurrences are generally linked to changes in the biology of the agent, host, environment, and their vectors. Such insights must be understood and applied within the framework of the concept of “One Health” [17]. The transmission of EMCV to pigs can occur through direct contact with infected animals. However, due to the short period of virus excretion, this route is less likely to introduce the virus to new places [3]. In Italy, a reported seasonal pattern of EMCV infection in breeding farms was reported and was attributed to the rodent tendency to inhabit the facilities during colder seasons [11]. These rodents (mice and rats) are considered the reservoir for EMCV, and they can excrete the virus for up to 29 days. Therefore, the presence of infected rodents plays an important role in the spread of the virus [18]. In the cases herein, a comparison of the sequence from the swine with the sequence from the rat revealed a nucleotide identity of 99.57%. This strongly suggests that rats were the source of the outbreaks, and they probably introduced the virus to the farms, thus infecting the pigs.

In our study, it was observed unusually high-mortality rates in swine, reaching 9%–10% of the batch, which typically do not exceed 2% [19]. This can be attributed to the introduction of a new pathogen into the farm, resulting in the absence of immunity against the virus. Additionally, the high pathogenicity of the EMCV, causing acute myocarditis, likely contributed to the increased mortality rates due to sudden death [18, 20]. After the diagnosis of EMCV infection was established on the farms, the company was recommended to reinforce rodent control measures, which yielded positive results, and the cases decreased thereafter. A similar scenario was observed in another recent report in Italy [20].

Pigs can be infected by EMCV at any stage of their life, with symptomatic and asymptomatic infections. In pigs at the nursery and growing–finishing stages, infections are more common and can cause sudden death; furthermore, when transmission is vertical, death may occur in the first days of life. Adult pigs may carry the infection without showing any apparent signs, while others may present reproductive failure, characterized by abortion, mummified and stillborn fetuses [3]. In the cases presented in this work, the affected pigs were in the growing–finishing phase, and infection by EMCV resulted in sudden death, as reported in the literature. Nevertheless, some clinical signs (trembling, squealing, and dyspnea) were observed, similar to the signs reported in other studies [20].

The gross and microscopic findings in the heart showed a characteristic pattern of viral myocarditis (mononuclear inflammation) associated with marked dystrophic mineralization. Although these microscopic findings are commonly seen in EMCV infection, it is important to consider some differential diagnoses, including *Aphtovirus* infection, *Parvovirus* infection, and vitamin E/selenium deficiency [20, 21]. The mentioned viral infections typically do not present mineralization, allowing them to be ruled out. However, mineralization is a typical finding of vitamin E/selenium deficiency, which was excluded as a cause due to the positive RT‒PCR test for EMCV. Although concurrent disease may occur, mineral dosage was not performed, as this situation is considered unlikely, since there was no evidence of hepatocellular necrosis or hemorrhage in the heart within the sections examined.

The name EMCV comes from the initial association of the virus with inflammation in both the brain and heart [5]. However, over time, it became evident that encephalitis was more frequently observed in experimentally infected mice and swine fetuses. In the present report, the examination of the brain was unremarkable, aligning with previous reports indicating that encephalitis is not a consistent feature of natural EMCV infection in pigs at the growing–finishing phase [21].

To confirm the diagnosis of EMCV infection, it is essential to detect the virus in the affected pigs. This can be achieved through various methods, including virus isolation, serological tests, or molecular methods [3]. In this study, the diagnosis was confirmed using RT‒PCR for the detection of the EMCV VP1 and RdRp genes. Such results align with the findings of previous studies that used similar molecular techniques for diagnosis [6, 18].

## 4. Conclusions

The detection of EMCV in Brazil after a gap of 38 years marks the third occurrence of the virus in the country. The outbreaks reported have been characterized by sudden death in growing–finishing pigs. Upon gross and microscopic examination, the main findings were myocardial necrosis associated with mononuclear inflammation, dystrophic mineralization, fibrosis, and signs of heart failure. The pig samples (liver, heart, and lymph node) and rat samples (brain and feces) tested positive in RT‒PCR against EMCV VP1 and RdRp, confirming the diagnosis after sequencing. The genetic characterization has shown that the Brazilian strains are closely related to Peruvian strains, providing the first genetic information about EMCV in Brazil. This represents a significant finding and underscores the importance of continuous monitoring and research to understand and manage viral infections in swine populations.

## Data Availability

The data that support the findings of this study are available from the corresponding author upon reasonable request.

## Supplementary Materials

Supplementary Materials Table S1: samples tested for encephalomyocarditis virus by RT‒PCR for RNA-dependent RNA polymerase (RdRp).
