# Supplementary material for: Reemerging of Encephalomyocarditis Virus in Pigs in Brazil: Pathological and Viral Characterization
Source: Transbound Emerg Dis. 2023 Dec 7;2023:6582778. doi: 10.1155/2023/6582778 (PMC12016915; doi:10.1155/2023/6582778)
Supplement: Supplementary Materials — Table S1: samples tested for encephalomyocarditis virus by RT‒PCR for RNA-dependent RNA polymerase (RdRp). [file 6582778.f1.docx]

**Supplementary Material**

**Re-emerging of encephalomyocarditis virus in pigs in Brazil: pathological and viral characterization**

**Anderson H. Gris, Raquel S. Alves, Laura J. Camargo, Letícia F. Baumbach, Jean C.O. Menegatt, Emanoelly M.S. Silva, Fernanda F. Perosa, Rafael P. Lima, Marianna Bertolini, Aparecida T.L. Fiúza, Tatiane T. N. Watanabe, Cláudio W. Canal, and David Driemeier**

**Supplementary Table 1:** Samples tested for encephalomyocarditis virus by RT‒PCR for RNA-dependent RNA polymerase (RdRp).

| **Animal** | **Origin** | **Lymph node** | **Heart** | **Liver** | **Feces** | **Brain** |
| --- | --- | --- | --- | --- | --- | --- |
| Pig 1-4 | Farm A and B | NC | NC | Positive* | NC | NC |
| Pig 5 | Farm A | Negative | Negative | NC | NC | NC |
| Pig 6 | Farm B | Positive | Positive | NC | NC | NC |
| Pig 7 | Farm A | Positive | Positive | NC | NC | Negative |
| Rat 1-5 | Farm A | Negative | Negative | NC | Positive* | Positive* |
| Rat 6-10 | Farm A | Negative | Negative | NC | Negative | Negative |

Positive*: Tested in pool. NC: Not collected.
